# Meta-Analysis of the Safety and Efficacy of α-Adrenergic Blockers for Pediatric Urolithiasis in the Distal Ureter

**DOI:** 10.3389/fped.2022.809914

**Published:** 2022-04-15

**Authors:** Fengze Sun, Xingjun Bao, Dongsheng Cheng, Huibao Yao, Kai Sun, Di Wang, Zhongbao Zhou, Jitao Wu

**Affiliations:** ^1^Department of Urology, Yantai Yuhuangding Hospital, Qingdao University, Yantai, China; ^2^The Second Clinical Medical College, Binzhou Medical University, Yantai, China; ^3^Department of Pharmacy, Yantai Yuhuangding Hospital, Qingdao University, Yantai, China; ^4^Department of Urology, Beijing Tiantan Hospital, Capital Medical University, Beijing, China

**Keywords:** alpha-adrenergic blockers, tamsulosin, silodosin, doxazosin, pediatric urolithiasis, meta-analysis, distal ureteral stones, randomized controlled trials

## Abstract

**Objective:**

Pediatric urolithiasis is a common condition, and medical expulsive therapy has grown to be accepted by many parents. We carried out a meta-analysis to identify the efficacy and safety of α-adrenergic blockers for the treatment of pediatric urolithiasis.

**Methods:**

We identified related articles from the PubMed, Embase, and Cochrane Library databases. All published randomized controlled trials (RCTs) describing the use of α-adrenergic blockers and placebo treatment for pediatric distal urolithiasis were involved. The outcomes included stone expulsion rate, stone expulsion time, pain episodes, need for analgesia, adverse events, and related subgroup analyses.

**Results:**

A total of nine RCTs were involved in our study, including 586 patients. We found that α-adrenergic blockers could significantly increase the rate of stone expulsion [odds ratio (OR), 3.49; 95% confidence interval (CI), 2.38–5.12; *p* < 0.00001], reduce the stone expulsion time [mean difference (MD), −5.15; 95% CI, −8.51 to −1.80; *p* = 0.003], and decrease pain episodes (MD, −1.02; 95% CI, −1.33 to −0.72; *p* < 0.00001) and analgesia demand (MD, −0.92; 95% CI, −1.32 to −0.53; *p* < 0.00001) but had a higher incidence of side effects (MD, 2.83; 95% CI, 1.55 to 5.15; *p* = 0.0007). During subgroup analyses, different medications (tamsulosin, doxazosin, and silodosin) also exhibited better efficiencies than placebo, except for doxazosin, which showed no difference in expulsion time (MD, −1.23; 95% CI, −2.98 to 0.51; *p* = 0.17). The three kinds of α-adrenergic blockers also appeared to be better tolerated, except for tamsulosin with its greater number of adverse events (MD, 2.85; 95% CI, 1.34 to 6.03; *p* = 0.006). Silodosin led to a better expulsion rate than tamsulosin (OR, 0.42; 95% CI, 0.20 to 0.92; *p* = 0.03). In addition, α-adrenergic blockers increased the stone expulsion rate regardless of stone size and decreased the expulsion time of stones measuring <5 mm (MD, −1.71; 95% CI, −2.91 to −0.52; *p* = 0.005), which was not the case for stones measuring >5 mm in expulsion time (MD, −3.61; 95% CI, −10.17 to 2.96; *p* = 0.28).

**Conclusion:**

Our review suggests that α-adrenergic blockers are well-tolerated and efficient for treating pediatric distal urolithiasis. We also conclude that silodosin is the best choice of drug, offering a better expulsion rate, but it remains to be evaluated further by future studies.

## Introduction

The occurrence of urolithiasis in children has become more and more frequent, with an incidence of about 0.1–5% (1–3). The reasons for urinary stone formation in children are various and include metabolic, environmental, and nutritional factors (4, 5). The typical presentations of urolithiasis are hematuria, dysuria, and pain in older children, whereas younger children present with non-specific symptoms like irritability (6).

The appropriate treatment for urolithiasis is generally selected according to stone size, location, and composition and the urinary tract anatomy (7). Over time, the main surgical technique has also evolved from open stone surgery to minimally invasive procedures with the development of endoscopic equipment (8). Medical expulsive therapy (MET) has also been accepted as initial management for small distal ureteric stones to avoid the risk of anesthesia and associated costs (9, 10), and α-adrenergic blockers are the preferred medication choice in this context (11).

In recent years, α-adrenergic blockers have been recommended for the treatment of distal ureter stones (12). Many studies have shown that α-adrenergic blockers achieve great success in both the spontaneous stone expulsion rate and time in adults (13–18). In addition, α-adrenergic blockers have also been applied to treat urolithiasis in children as MET (19), but few strong evidence-based studies on the feasibility of using α-adrenergic blockers in the treatment of urolithiasis in children exist to date.

In this systematic review and meta-analysis, we assessed the efficacy of α-adrenergic blockers as a medical treatment for distal ureteral stones in pediatric patients using data from published randomized controlled trials (RCTs).

## Methods

### Search Strategy

This analysis followed the Preferred Reporting Items for Systematic Reviews and Meta-analyses guideline (20). RCTs covering α-adrenergic blockers in the treatment of pediatric distal urolithiasis were collected by three authors from the PubMed (1997 to November 2021), Embase (1997 to November 2021), and Cochrane Library (1997 to November 2021) literature databases. Table 1 summarizes the search strategy using the Populations, Interventions, Comparators, Outcomes, and Study designs. The search terms used were as follows: “alpha-adrenergic blockers,” “distal ureter,” “ureteral stone,” “calculi,” “urolithiasis,” “children,” “pediatric,” and “randomized controlled trials.” We reviewed all search results to confirm the availability of studies and to extract further information. The references of related articles were also searched for eligible studies.

**Table 1:** Search strategy according to populations, interventions, comparators, outcomes, and study designs (PICOS).

	**Population**	**Intervention**	**Comparator**	**Outcomes**	**Study design**
Inclusion criteria	Age <18 years old Stone size <12 mm Distal ureteral stone	α-adrenergic blockers (including tamsulosin, silodosin, doxazosin)	placebo	Stone expulsion rate Expulsion time Pain episodes Need for analgesia Adverse events. Headache. Nasal congestion	Randomized Controlled Trials
Exclusion criteria	Age more than 18 years old Stone size more than 12 mm Multiple or bilateral ureteric stones Single kidney Abnormal renal function Marked hydronephrosis Urinary tract infection Urinary tract anomalies Voiding dysfunction	Not performed	Not performed	Subjective feeling score scale such as visual analog scale (VAS) or treatment satisfaction questionnaires (TSQ).	Letters, comments, reviews, qualitative studies

### Inclusion Criteria

Studies were included in this analysis if they met the following criteria: 1) the study analyzed the efficiency of α-adrenergic blockers and placebo treatment in pediatric distal urolithiasis; 2) a full-text version of the article could be obtained; 3) the study was an RCT and provided complete and accurate data, including its sample size and the valuable results of each indicator; 4) patients were <18 years of age. The related details of inclusion criteria are shown in Table 1. If a study was published in multiple journals or at different times, the most comprehensive version of the study was included in this investigation. If the same group of participants was studied by one group of researchers in several experiments, all studies were included. Compared with retrospective studies, RCTs have stricter inclusion and exclusion criteria.

### Quality Assessment

The quality of all included RCTs was evaluated using the *Cochrane Handbook* (21). The quality of individual studies was determined according to its assessment methods, including patient allocation, concealment of allocation, and blinding method. Each included study was assessed using the guidelines published in the *Cochrane Handbook for Systematic Reviews of Interventions*, version 5.4.0 (22). All studies were classified based on the following quality assessment criteria: (+) the study met all quality criteria and had a low risk of bias; (?) the study met most quality criteria and had a moderate risk of bias; or (–) the study met few quality criteria and had a high risk of bias. Different opinions about the classifications of studies were solved by discussion among all authors.

### Data Extraction

We collected valuable information from all included articles, including the name of the first author, the publication time, the study type, the capacity of the study sample, the treatment of patients, and data of study outcomes (i.e., stone expulsion rate, expulsion time, adverse events, pain episodes, analgesia requirement, and stone size). The main outcome was the stone expulsion rate, and the secondary outcome was the stone expulsion time; however, other outcomes like pain episodes and analgesia demand were also analyzed, and the safety of different medications was identified by adverse events. The stone expulsion rate was defined by the ratio of pediatric patients with complete expulsion and incomplete expulsion, which was tested using plain kidney ureter bladder, non-contrast CT, and ultrasonography. The stone expulsion time was defined as the time to stone passage, which was confirmed by visual observation of some or all of the stone.

### Statistical Analysis and Meta-Analysis

The data of this study were statistically analyzed using the Review Manager software (version 5.4.0; Cochrane Collaboration, London, UK) (22). Changes in the stone expulsion time, stone expulsion rate, pain episodes, and adverse events were analyzed to declare the effect of α-adrenergic blockers in the treatment of pediatric urolithiasis. Continuous data were analyzed by the mean difference (MD), and dichotomous data were evaluated by odds ratios (ORs) with 95% confidence intervals (95% CIs) (23). A fixed-effects model was applied if the study was deemed to be homogenous; if the *p* > 0.05, then a random-effects model was used. Inconsistent results were analyzed with the *I*^2^ statistic. If the *p* < 0.05, the results were considered to indicate statistical significance.

## Results

### Characteristics of Included Studies

**Figure 1:**
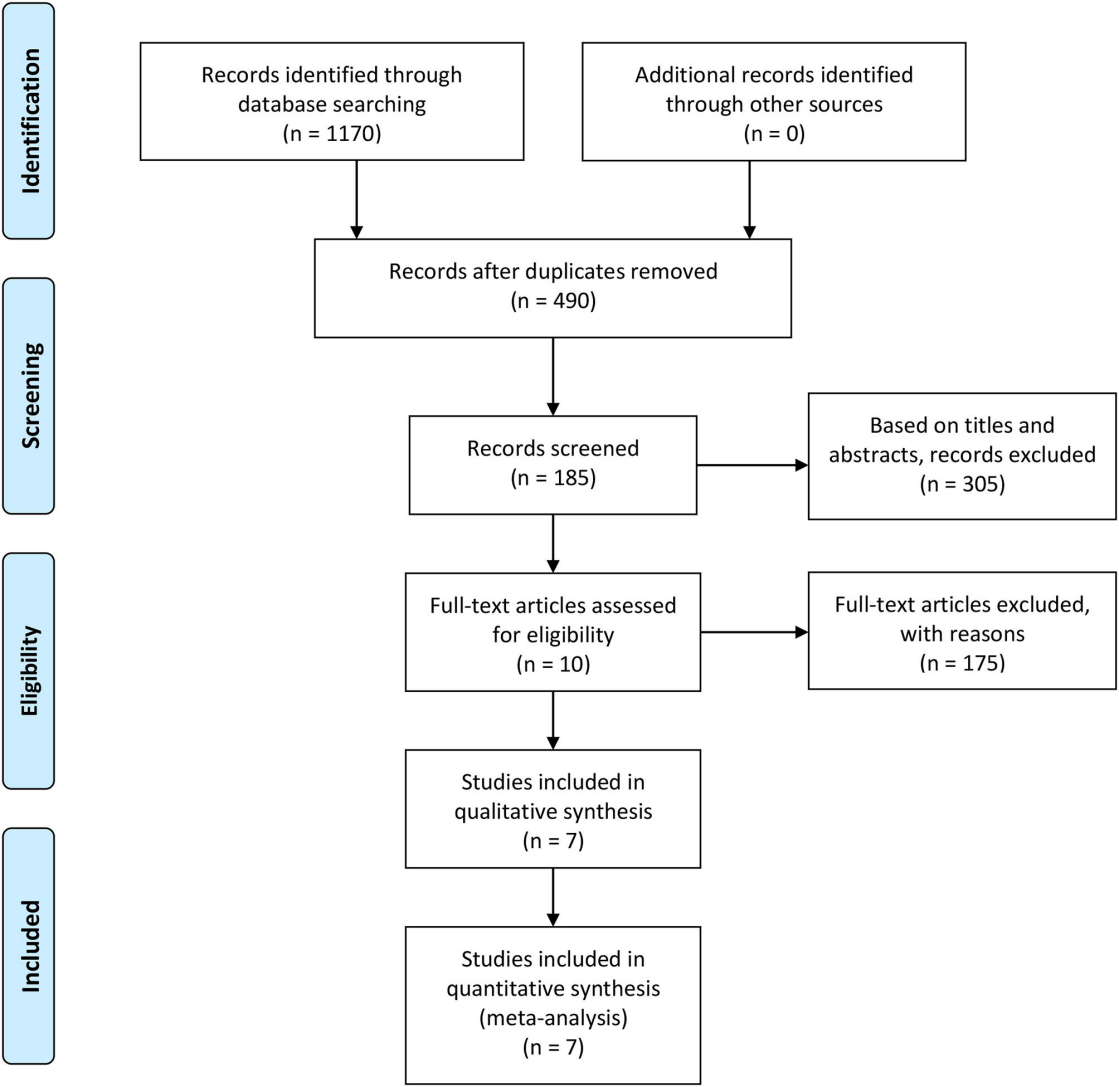
PRISMA flow diagram.

A total of 185 articles found to meet the inclusion criteria after their titles and abstracts were read were included. After reviewing the tables and figures in each article, however, 175 articles were excluded due to a lack of useful data. Then, when we reviewed the remaining 10 articles, 3 articles were further excluded because of their article designs. Finally, 7 high-quality articles were included in our review (24–30). Because 2 intervention groups were set in 2 articles, we regarded these 2 articles as 4 individual RCTs (28, 30). The 2 different RCTs in each study were marked as a and b. The details of the study selection process are shown in Figure 1, and the characteristics and features of 9 included RCTs are presented in Table 2.

**Table 2:** Study and patient characteristics.

**References**	**Country**	**Design**	**Therapy in** **experiment group**	**Therapy in** **control group**	**Simple** **size**		**Loss to** **follow-up**	**Method**	**Time of** **therapy** **(weeks)**	**Dosage (mg)**	**Inclusion** **population**	**Statistical** **analysis**	**ITT** **analysis**
					**Trial**	**Control**							
Aydogdu et al. (24)	Turkey	RCT	Doxazosin	Analgesics	19	20	0	Oral	3	0.03 mg/kg/d	Patients 2–14 y with DUS ≤ 10 mm	ANCOVA	No
Mokhless et al. (25)	Egypt	RCT	Tamsulosin	Analgesics	33	28	0	Oral	4	0.4 mg/d	Patients 2–15 y with DUS ≤ 12 mm	ANCOVA	No
Erturhan et al. (26)	Turkey	RCT	Doxazosin	Analgesics	24	21	0	Oral	3	0.03 mg/kg/d	Patients 3–15 y with DUS ≤ 10 mm	ANCOVA	No
Aldaqadossi et al. (27)	Egypt	RCT	Tamsulosin	Analgesics	33	34	4	Oral	4	0.4 mg/d	Patients 2–15 y with DUS ≤ 10 mm	ANCOVA	No
Fahmy et al. (28)^a^	Egypt	RCT	Tamsulosin	Analgesics	30	30	2	Oral	4	8 mg/d	Patients 5.8–18 y with DUS ≤ 10 mm	ANCOVA	No
Fahmy et al. (28)^b^	Egypt	RCT	Silodosin	Analgesics	30	30	0	Oral	4	0.4 mg/d	Patients 5.8–18 y with DUS ≤ 10 mm	ANCOVA	No
Elgalaly et al. (29)	Egypt	RCT	Silodosin	Analgesics	18	19	2	Oral	4	4 mg/d	Patients <18 y with DUS ≤ 10 mm	ANCOVA	No
Soliman et al. (30)^a^	Egypt	RCT	Tamsulosin	Analgesics	63	63	15	Oral	4	0.4 mg/d	Patients 6–14 y with DUS ≤ 10 mm	ANCOVA	No
Soliman et al. (30)^b^	Egypt	RCT	Silodosin	Analgesics	63	63	14	Oral	4	4 mg/d	Patients 6–14 y with DUS ≤ 10 mm	ANCOVA	No

*RCT, random controlled trials; ANCOVA, analysis of covariance; ITT, intention-to-treat*.

### The Quality of Eligible Studies

**Figure 2:**
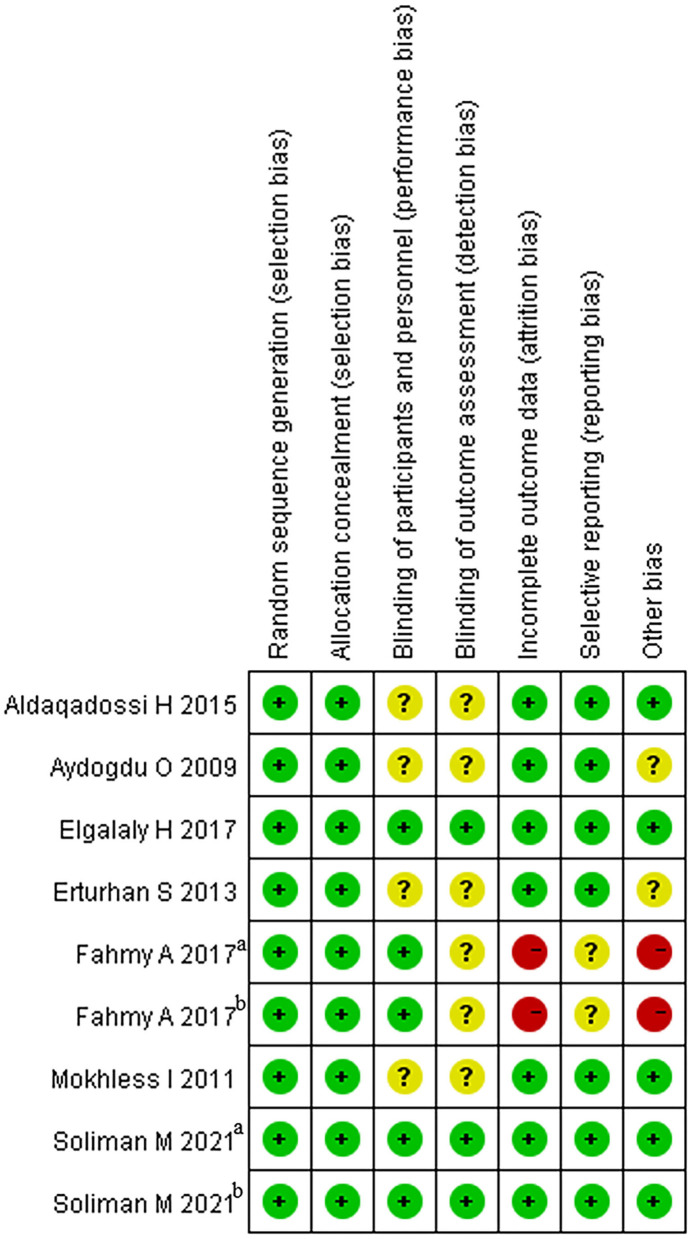
The risk of bias graph.

**Figure 3:**
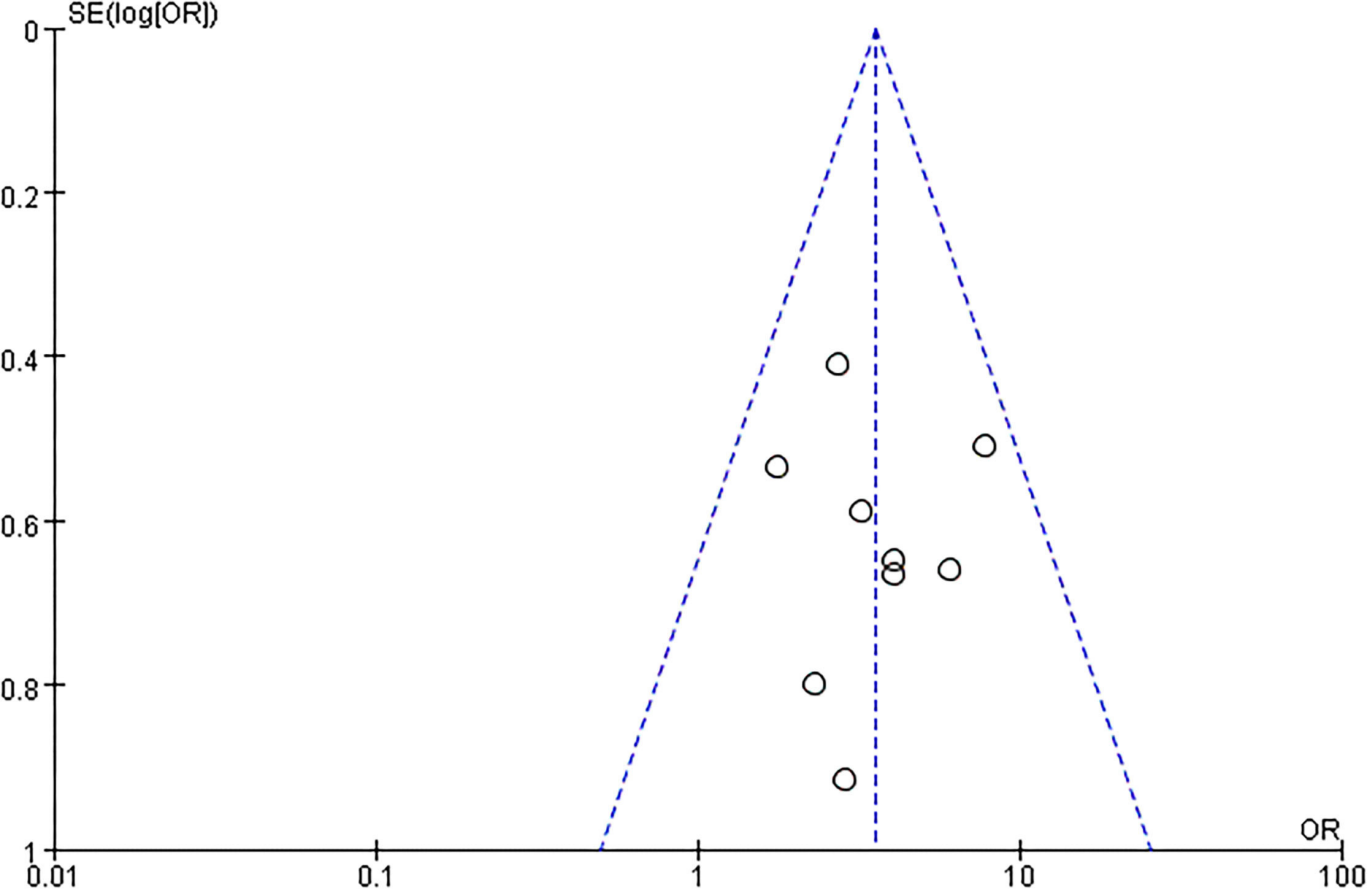
The risk of bias graph.

The articles included in our study were all RCTs, including 5 that were blinded RCTs (28–30). As a blinding method was not mentioned in 4 articles (24–27), the grade of the blinding method was “?” Two RCTs offered less useful data, so its quality grade was “–” (28). Details are shown in Figure 2. The capacity of each RCT was calculated. The funnel plot showed that the publication bias of all studies was not identifiable in this study (Figure 3).

### Efficacy

#### Stone Expulsion Rate

**Figure 4:**
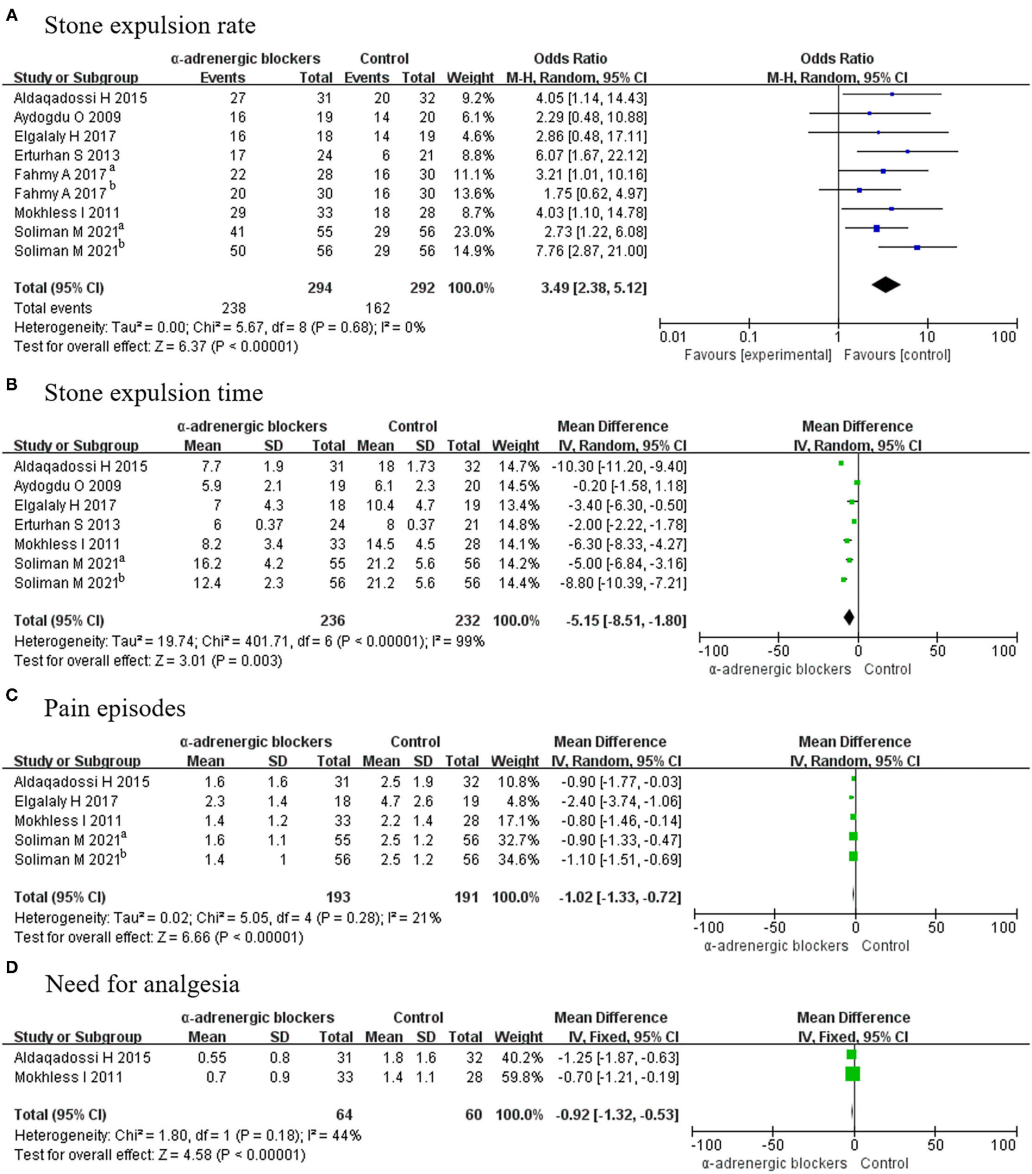
Forest plots showing the result of **(A)** stone expulsion rate, **(B)** stone expulsion time, **(C)** pain episodes, and **(D)** need for analgesia. M–H, Mantel–Haenszel; CI, confidence interval; df, degrees of freedom.

All included articles (9 RCTs) contained data on the stone expulsion rate from 586 patients, including 294 patients in the experimental group and 292 participants in the control (placebo) group. We deemed the trials to be homogenous given that *p* > 0.05, so a fixed-effects model was selected for analysis and showed that the OR was 3.49 and the 95% CI was 2.38–5.12 (*p* < 0.00001) (Figure 4A). The result suggested that α-adrenergic blockers could improve the stone expulsion rate more effectively than placebo.

#### Stone Expulsion Time

Five articles covering 6 RCTs, in which a total of 236 experimental patients and 232 control participants were considered, reported on the stone expulsion time. A random-effects model was chosen to analyze these data because *p* < 0.05. The MD was −5.15, with a 95% CI of −8.51 to −1.80 (*p* = 0.003) (Figure 4B). It was concluded that α-adrenergic blockers had a shorter stone expulsion time than placebo.

#### Pain Episodes

Four articles covering 5 RCTs offering a total of 384 patients, including 193 patients in the experimental group and 191 participants in the control group, reported on pain outcomes. A fixed-effects model was chosen for analysis and revealed that the MD was −1.02 and the 95% CI was −1.33 to −0.72 (*p* < 0.00001) (Figure 4C). This result meant that α-adrenergic blockers experienced less pain than placebo.

#### Need for Analgesia

Only 2 RCTs offering 124 patients—including 64 experimental patients and 60 control participants—reported on the need for analgesia, and a fixed-effects model indicated that the experimental group required less placebo (MD, −0.92; 95% CI, −1.32 to −0.53; *p* < 0.00001) (Figure 4D).

### Safety

#### Adverse Events

**Figure 5:**
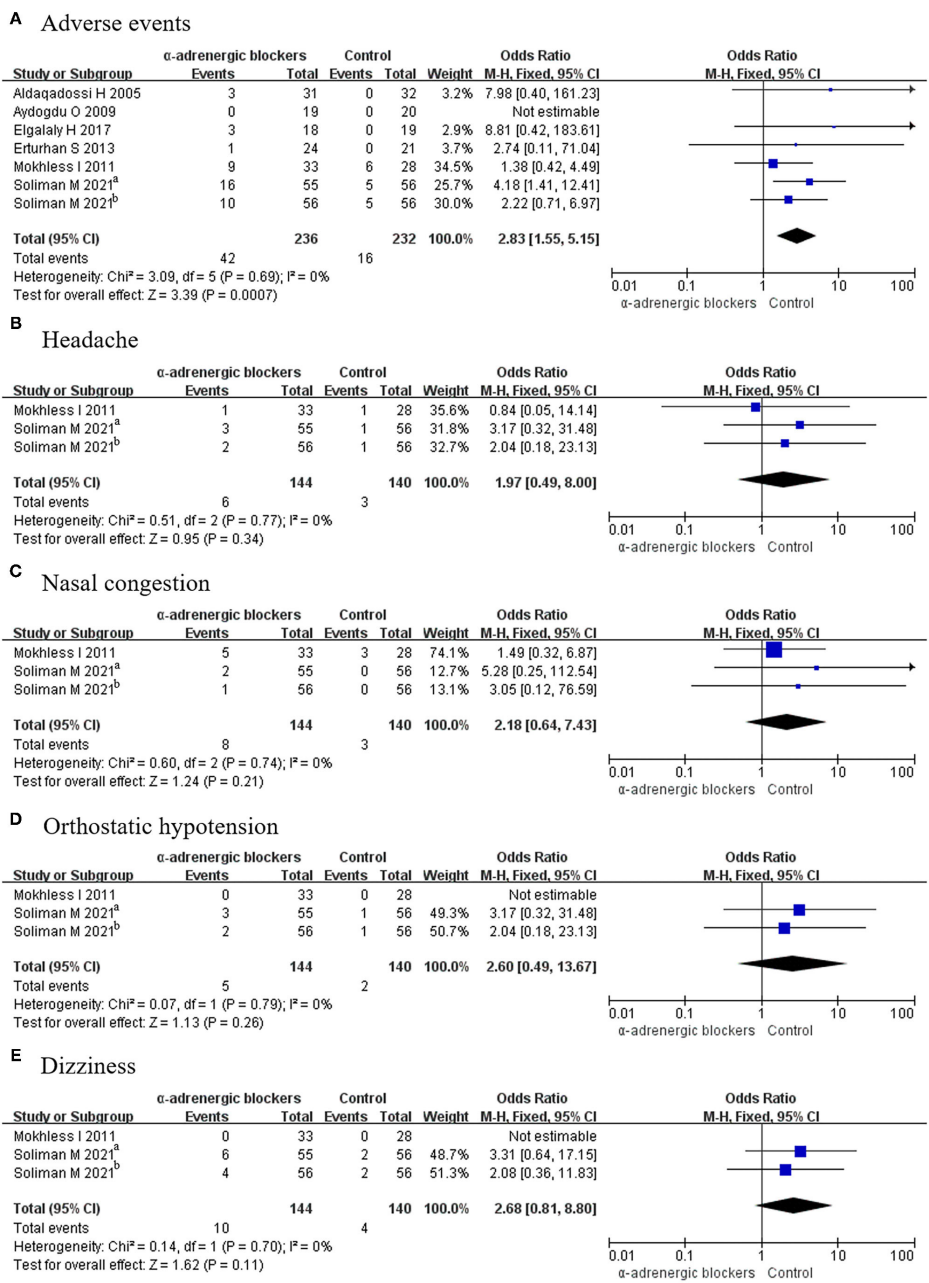
Forest plots showing the result of **(A)** adverse events, **(B)** headache, **(C)** nasal congestion, **(D)** orthostatic hypotension, and **(E)** dizziness. M–H, Mantel–Haenszel; CI, confidence interval; df, degrees of freedom.

Six articles covering 7 RCTs, in which 236 patients received α-adrenergic blockers and 232 patients only received placebo, described the related details of adverse events. Because *p* > 0.05, a fixed-effects model was selected, and it was found that the OR was 2.83 and the 95% CI was 1.55–5.15 (*p* = 0.0007) (Figure 5A), which meant that the α-adrenergic blockers led to more adverse events than did placebo.

#### Headache

Two articles covering 3 RCTs offered data on headache from 284 patients, including 144 patients in the experimental group and 140 participants in the control group. Given that *p* > 0.05, a fixed-effects model was used to compare the rate of headache between the 2 groups, and the OR was 1.97 and the 95% CI was 0.49–8.00, with a *p* > 0.05 (Figure 5B). This result signified that there was no difference in the rate of headache between the groups.

#### Nasal Congestion

Because *p* > 0.05, a fixed-effects model was used to analyze the rate of nasal congestion. A total of 3 RCTs were considered, among which 144 patients accepted α-adrenergic blockers and 140 patients accepted placebo. The results showed that the OR was 2.18 and the 95% CI was 0.64–7.43 (*p* > 0.05) (Figure 5C). This indicated that there was no difference in the occurrence of nasal congestion between the 2 groups.

#### Orthostatic Hypotension

Three RCTs discussed orthostatic hypotension, which occurred in 5 patients in the experimental group and 2 participants in the control group. Given that *p* > 0.05, a fixed-effects model was selected, and there was no difference between the groups, as the OR was 2.60 and the 95% CI was 0.49–13.67 (*p* > 0.05) (Figure 5D).

#### Dizziness

Dizziness was described in 3 RCTs involving 284 patients. A fixed-effects model was chosen given that *p* > 0.05, and the OR was 2.68 and the 95% CI was 0.81–8.80 (*p* > 0.05). No difference was found between α-adrenergic blockers and placebo (Figure 5E).

### Subgroup Analysis

#### Different Medications

In this study, 3 kinds of α-adrenergic blockers, including tamsulosin, silodosin, and doxazosin, were used to treat pediatric urolithiasis. Subgroup analysis was performed in view of these different drugs.

#### Efficiency

##### Stone Expulsion Rate

**Figure 6:**
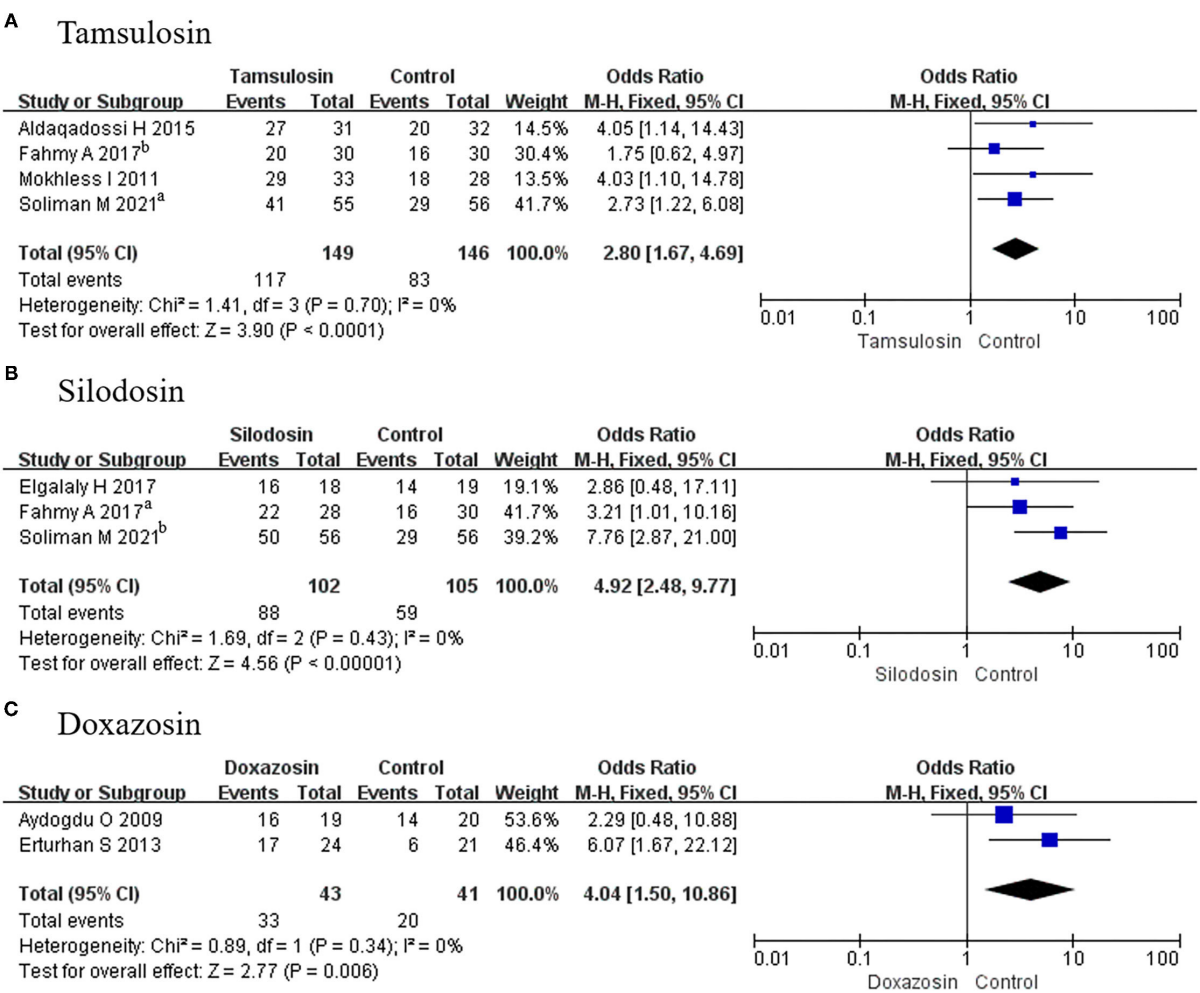
Forest plots showing the result of **(A)** subgroup analyses of tamsulosin in stone expulsion rate, **(B)** subgroup analyses of silodosin in stone expulsion rate, and **(C)** subgroup analyses of doxazosin in stone expulsion rate. M–H, Mantel–Haenszel; CI, confidence interval; df, degrees of freedom.

In this assessment, 4 RCTs using tamsulosin, 3 RCTs using silodosin, and 2 RCTs using doxazosin were absorbed. The expulsion rate associated with tamsulosin was greater than that of placebo (OR, 2.80; 95% CI, 1.67–4.69; *p* < 0.0001) (Figure 6A). There was a significant difference in this parameter between silodosin and placebo (OR, 4.92; 95% CI, 2.48–9.77; *p* < 0.00001) (Figure 6B), and the same result was also found for doxazosin (OR, 4.04; 95% CI, 1.50–10.86; *p* = 0.006) (Figure 6C).

##### Stone Expulsion Time

**Figure 7:**
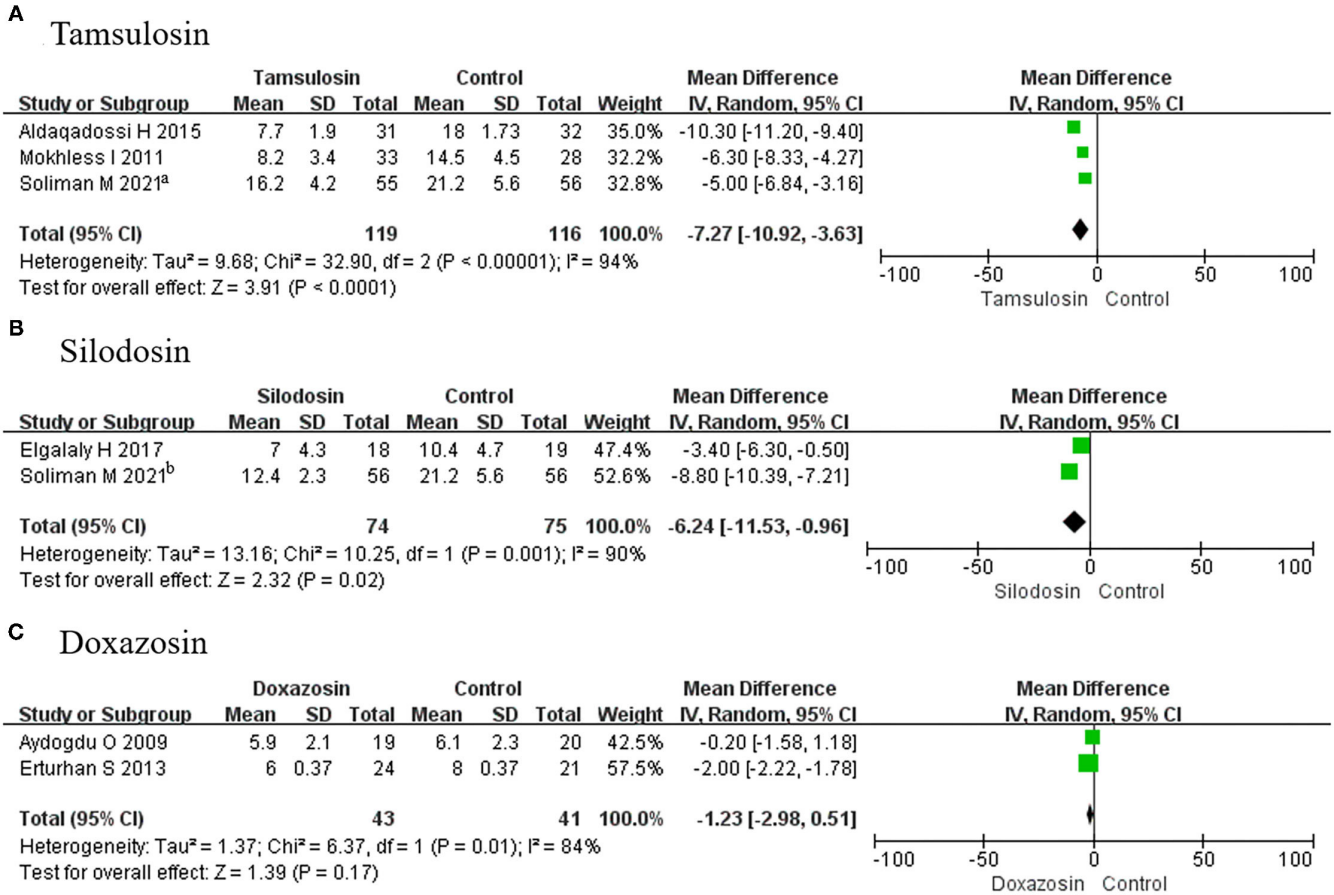
Forest plots showing the result of **(A)** subgroup analyses of tamsulosin in stone expulsion time, **(B)** subgroup analyses of silodosin in stone expulsion time, and **(C)** subgroup analyses of doxazosin stone in expulsion time. M–H, Mantel–Haenszel; CI, confidence interval; df, degrees of freedom.

In this analysis, 2 RCTs using tamsulosin, 2 RCTs using silodosin, and 2 RCTs using doxazosin were considered. It was found that the stone expulsion time was less for tamsulosin (MD, −7.27; 95% CI, −10.92 to −3.63; *p* < 0.0001) (Figure 7A) and silodosin (MD, −6.24; 95% CI, −11.53 to −0.96; *p* = 0.02), compared with placebo (Figure 7B). However, it was not different for doxazosin (MD, −1.23; 95% CI, −2.98 to 0.51; *p* = 0.17) (Figure 7C).

##### Pain Episodes

**Figure 8:**
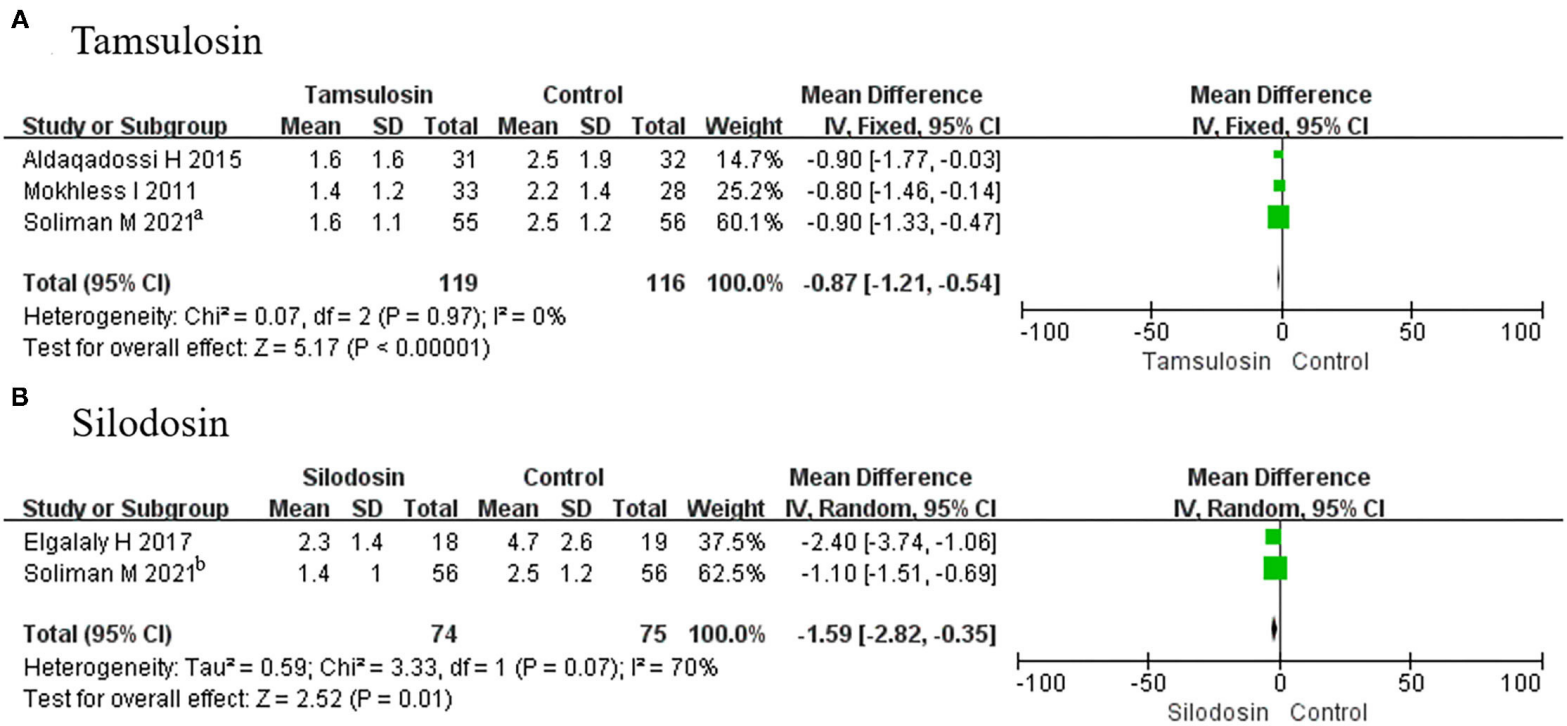
Forest plots showing the result of **(A)** subgroup analyses of tamsulosin in pain episodes and **(B)** subgroup analyses of silodosin in pain episodes. M–H, Mantel–Haenszel; CI, confidence interval; df, degrees of freedom.

A total of 3 RCTs using tamsulosin and 2 RCTs using silodosin were included in this group. We found that pain episodes were less frequent among patients receiving tamsulosin (MD, −0.87; 95% CI, −1.21 to −0.54; *p* < 0.00001) (Figure 8A) and silodosin (MD, −1.59; 95% CI, −2.82 to −0.35; *p* = 0.01) (Figure 8B), compared with placebo.

##### Adverse Events

**Figure 9:**
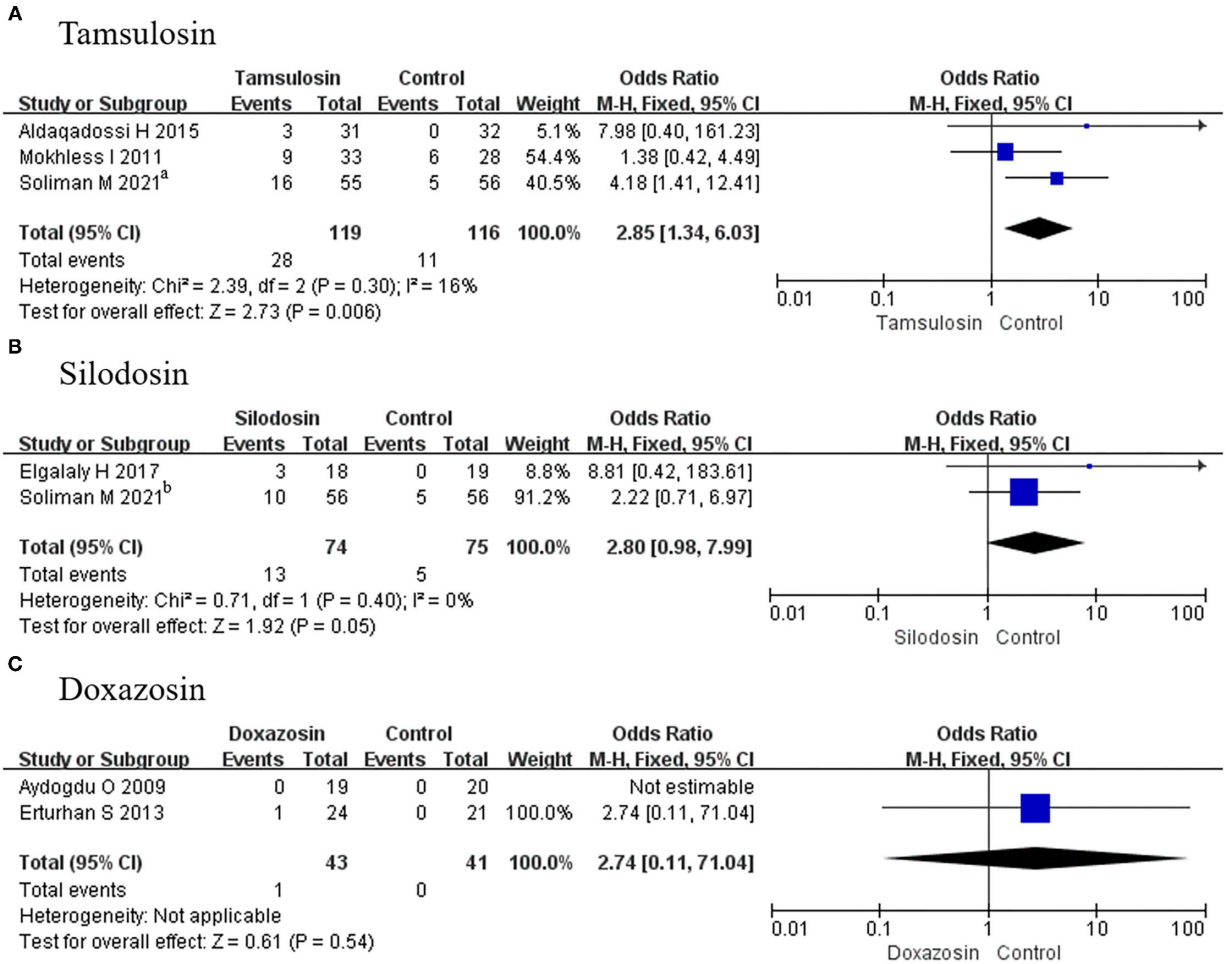
Forest plots showing the result of **(A)** subgroup analyses of tamsulosin in adverse events, **(B)** subgroup analyses of silodosin in adverse events, and **(C)** subgroup analyses of doxazosin in adverse events. M–H, Mantel–Haenszel; CI, confidence interval; df, degrees of freedom.

A total of 3 RCTs using tamsulosin and 2 RCTs using silodosin reported adverse events. Given that *p* > 0.05, fixed-effects models were adopted in the analysis of tamsulosin and silodosin, and we found a significant difference for tamsulosin (OR, 2.85; 95% CI, 1.34–6.03; *p* = 0.006) (Figure 9A), compared with placebo. In contrast, there was no difference recorded for silodosin (OR, 2.80; 95% CI, 0.98–7.99; *p* = 0.05) (Figure 9B) or doxazosin (MD, 2.74; 95% CI, 0.11–71.04; *p* = 0.54) (Figure 9C).

#### Tamsulosin and Silodosin

**Figure 10:**
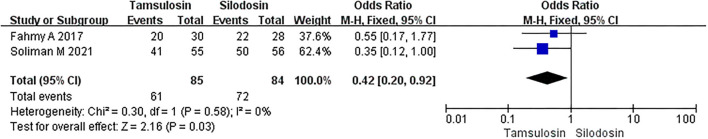
Forest plots showing the result of stone expulsion rate between tamsulosin and silodosin. M–H, Mantel–Haenszel; CI, confidence interval; df, degrees of freedom.

Two articles not only described the efficiency between α-adrenergic blockers and placebo but also offered a comparison of data for tamsulosin and silodosin. A total of 169 patients were involved in this subgroup analysis, and a fixed-effects model was used for analysis given that *p* > 0.05. Compared with tamsulosin, silodosin appeared to ensure a better expulsion rate, as the OR was 0.42 and the 95% CI was 0.20–0.92 (*p* = 0.03) (Figure 10).

#### Different Stone Sizes

**Figure 11:**
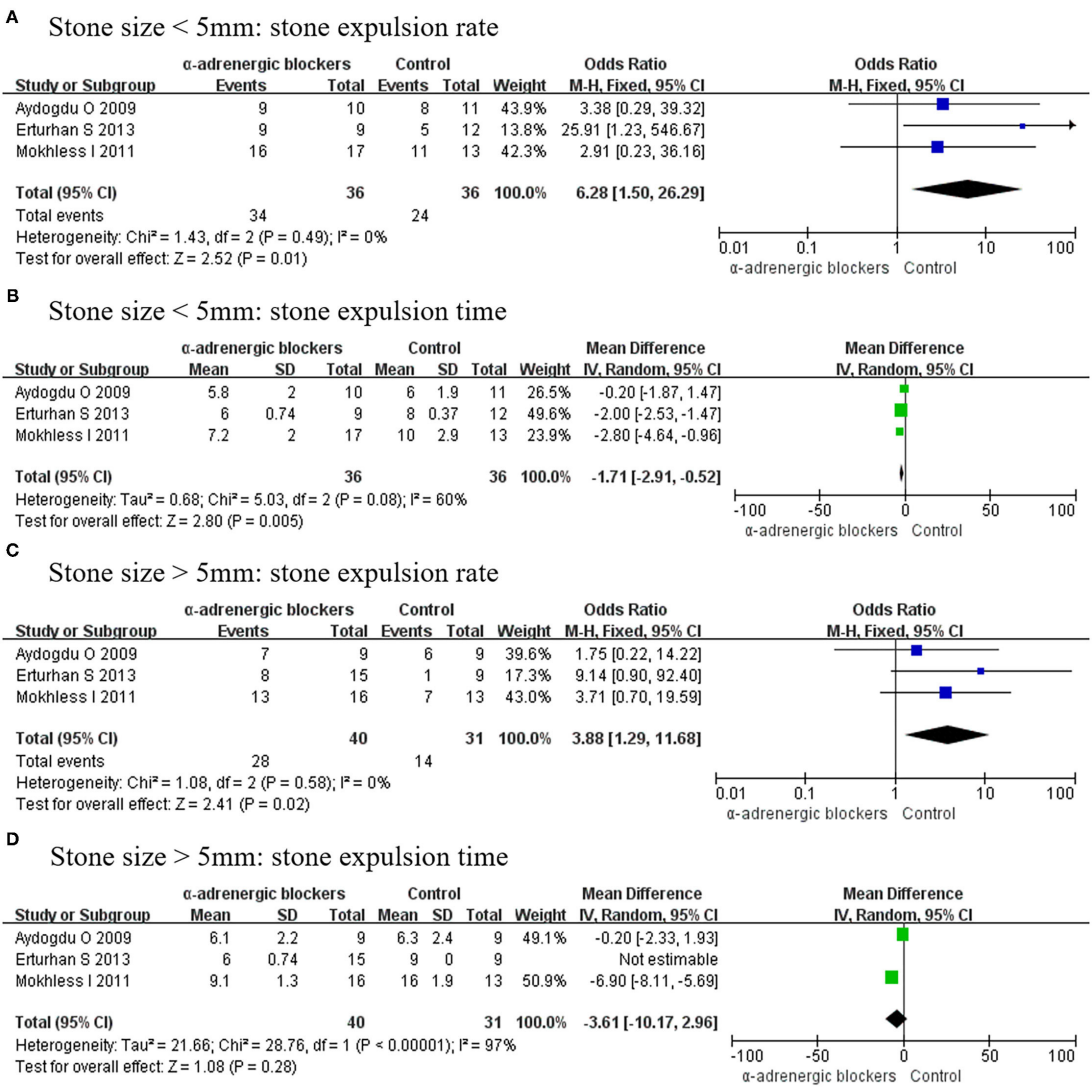
Forest plots showing the result of **(A)** stone expulsion rate in patients with stone size <5 mm, **(B)** stone expulsion time in patients with stone size <5 mm, **(C)** stone expulsion rate in patients with stone size >5 mm, and **(D)** stone expulsion time in patients with stone size >5 mm. M–H, Mantel–Haenszel; CI, confidence interval; df, degrees of freedom.

As different stone sizes were analyzed in 3 RCTs, stone size was divided into 2 groups of <5 and >5 mm. When the stone size was <5 mm, α-adrenergic blockers could increase the stone expulsion rate (OR, 6.28; 95% CI, 1.50–26.29; *p* = 0.01) (Figure 11A) and decrease the stone expulsion time (MD, −1.71; 95% CI, −2.91 to −0.52; *p* = 0.005) (Figure 11B). However, if the stone size was >5 mm, then α-adrenergic blockers could only increase the stone expulsion rate (OR, 3.88; 95% CI, 1.29–11.68; *p* = 0.02) (Figure 11C) and were useless for improving the stone expulsion time (MD, −3.61; 95% CI, −10.17 to 2.96; *p* = 0.28) (Figure 11D).

## Discussion

Pediatric urolithiasis happens more frequently in developing countries compared with developed countries (31). Generally, pediatric patients undergo treatment more than once given the high recurrence rate of urolithiasis. Given the cost and procedural risks, multiple medicines are used widely instead of surgery (32), including placebo, α-adrenergic blockers (e.g., tamsulosin, silodosin, doxazosin), calcium channel blockers (e.g., nifedipine), and other adjuvant medications (e.g., steroids or tolterodine). Analgesics could relieve pain to allow the body to expulse the stone autonomously, while α-adrenergic blockers can inhibit uncoordinated frequency and maintain propulsive contractions to accelerate stone expulsion and reduce pain levels (33). However, the efficacy of α-adrenergic blockers for pediatric urolithiasis in the distal ureter was not clear.

In our meta-analysis, we compared α-adrenergic blockers (tamsulosin, doxazosin, and silodosin) and placebo in the treatment of pediatric urolithiasis with a stone size of <12 mm. Among 9 RCTs including 294 patients in the experimental group and 292 participants in the control group, we recorded a significant efficiency of α-adrenergic blockers in treating pediatric urolithiasis. Notably, α-adrenergic blockers could improve the stone expulsion rate and decrease the expulsion time obviously, and the pain episodes of children receiving α-adrenergic blockers were fewer in number than those of children receiving placebo only, so the need for analgesics in the experimental group was less. In addition, we also carried out some subgroup analyses according to different medicines and stone sizes. Tamsulosin, silodosin, and doxazosin all showed immense efficiency in improving the expulsion rate, while both tamsulosin and silodosin showed a significant beneficial effect on expulsion time, but doxazosin showed no difference compared with placebo. Both tamsulosin and silodosin could reduce the frequency of pain episodes remarkably. Although it has been confirmed previously that silodosin leads to a better expulsion rate, shorter expulsion time, and fewer pain episodes in adult patients (34), in this study, we have compared tamsulosin and silodosin in pediatric patients for the first time, confirming again that silodosin had a better expulsion rate than tamsulosin. The explanation for this result was that the two main different subtypes of α-adrenergic receptors distributed in the human ureter, including α-1D and α-1A receptors, contribute to stone expulsion (35–37). Silodosin had an equal affinity to that of tamsulosin for the α-1D receptor subtype, but the affinity of silodosin for the α-1A receptor subtype is ~17-fold greater than that of tamsulosin (38). Therefore, it is no surprise that silodosin showed a better efficiency than tamsulosin and doxazosin in pediatric urolithiasis. In addition, during the subgroup analyses according to stone size, the expulsion rate and expulsion time were improved obviously in cases of stones measuring <5 mm, but the expulsion time was not different between the two groups for those measuring >5 mm. In summary, α-adrenergic blockers can enhance the stone expulsion efficiency, especially for smaller stones, which is a finding that contrasts with the current guidelines. The main reason for this discordant result may be the small sizes of the experimental and control groups.

In view of safety, a total of seven RCTs referred to the tolerance of α-adrenergic blockers. According to the collected data, adverse events were frequently observed in the experimental group. According to the subgroup analyses of different medicines, silodosin and doxazosin showed no differences compared with placebo, but tamsulosin appeared to trigger more side effects. When focusing only on common side effects of α-adrenergic blockers, including orthostatic hypotension, headache, dizziness, and nasal congestion, there were also no differences between the two groups.

A previous article including 3 RCTs and 2 retrospective cohort studies reported that MET could accelerate stone expulsion (39), but the inclusion criteria of this study were not limited to only treatment with α-adrenergic blockers (tamsulosin, doxazosin), and other medications, including calcium channel blockers or adjuvant medications, were also included. Furthermore, because of the limited available data, the conclusion in said article was not convincing, with a high degree of publication bias and an uncertain risk of significant bias. Overall, this previous study did not effectively conclude the effect of α-adrenergic blockers in pediatric urolithiasis. Two other articles have also reviewed the efficiency of α-adrenergic blockers (tamsulosin and doxazosin) in pediatric urolithiasis (40, 41). In the first study (40), only 3 RCTs were involved, including a total of 145 patients, and the results were limited by the scale of included trials with a high degree of publication bias, and the expulsion rate and pain episodes were analyzed without side effects analysis, which led to an incomplete result. A total of 406 patients from 4 RCTs and 1 cohort study were analyzed in the other review (41) but, owing to the still-larger number of patients included in our study, some contrasting results could be pointed out. Considering expulsion time, our study indicated that α-adrenergic blockers showed better efficiency than placebo, whereas this difference was not found in the aforementioned review. Moreover, tamsulosin showed more side effects compared with placebo in our study. Some other valuable information, like pain episodes, analgesia demand, and different stone sizes, was also analyzed in our study yet ignored in the previous one. The present study may therefore change some minds about drug selection in pediatric urolithiasis by modifying some inaccurate results in the literature and adding comparisons. We have also described the advantages of silodosin in pediatric urolithiasis compared with tamsulosin for the first time.

Importantly, there are some limitations that need to be considered in our study. First, our included articles were all from Turkey and Egypt, which could result in publication bias. We will focus in the future on publications from different regions of the world to broaden our conclusion. Second, the qualities of included studies were heterogeneous. Different randomization processes and blinding methods were accepted, and the measurement methods of outcomes in different studies were not all the same. In all, our results should be verified by further studies.

## Conclusion

Our study suggested that α-adrenergic blockers could promote stone expulsion efficiency in pediatric urolithiasis, especially for small stones measuring <5 mm, with a favorable safety profile. We also found that silodosin was the best medication choice, leading to a better expulsion rate, but it remains to be evaluated by further studies.

## Author Contributions

JW and ZZ designed the research, interpreted the data, and revised the paper. FS, XB, DC, HY, KS, and DW performed the data extraction and carried out the meta-analysis. FS drafted the paper. All authors approved the submitted and final versions.

## Funding

This work was supported by grants from the National Nature Science Foundation of China (Nos. 81870525 and 81572835) and Taishan Scholars Program of Shandong Province (No. tsqn201909199).

## Conflict of Interest

The authors declare that the research was conducted in the absence of any commercial or financial relationships that could be construed as a potential conflict of interest.

## Publisher's Note

All claims expressed in this article are solely those of the authors and do not necessarily represent those of their affiliated organizations, or those of the publisher, the editors and the reviewers. Any product that may be evaluated in this article, or claim that may be made by its manufacturer, is not guaranteed or endorsed by the publisher.
